# 1431. Evaluation of Drug-induced Liver Injury in Patients Receiving Anti-tuberculosis Medications and Subsequent Management

**DOI:** 10.1093/ofid/ofac492.1260

**Published:** 2022-12-15

**Authors:** Carina Acosta, Barbara Barsoum, Thien-Ly Doan

**Affiliations:** Long Island Jewish Medical Center, New Hyde Park, New York; Long Island Jewish Medical Center, New Hyde Park, New York; Long Island Jewish Medical Center, New Hyde Park, New York

## Abstract

**Background:**

For anti-tubercular medications, the risk of drug-induced liver injury (DILI) ranges from 5-33%. DILI may be asymptomatic or may present with fever, nausea, vomiting, anorexia, and lethargy. The objective of this review is to describe the incidence of hepatotoxicity occurring with anti-tuberculosis medications, management of DILI, and re-initiation of therapy.

**Methods:**

Adult patients prescribed pyrazinamide during their hospital stay at Long Island Jewish Medical Center were screened for inclusion criteria of DILI (liver enzymes ≥ 3x upper limit of normal [ULN] from 7/2017 to 2/2022. IRB classified this analysis as a quality improvement project. Using the electronic medical record, retrospective data was collected for those meeting criteria for DILI which included baseline demographics, concomitant hepatotoxic medications, laboratory values (e.g., alanine aminotransferase [ALT], aspartate aminotransferase [AST], timing of drug interruption, and sequence of re-initiation.

**Results:**

A total of 108 patients were screened and then 17 were included in the analysis. DILI was found in 16% of patients. The mean age was 57 years and 53% were female. None of the patients with liver injury had a history of hepatic disease, prior DILI, or alcohol consumption. Mean baseline laboratory values were total bilirubin 0.52 mg/dL, alkaline phosphatase 166 U/L, AST 54 U/L, and ALT 40 U/L. All patients were initiated on first-line treatment for drug-susceptible tuberculosis. Thirteen patients had concomitant hepatotoxic drugs (11 acetaminophen, 4 statins, 2 azole antifungals, 1 trimethoprim/sulfamethoxazole). Fourteen patients (82%) developed DILI where AST was ≥ 10 times the ULN. Drug interruption was necessary in 16 of the 17 patients (94%). Seven patients (58%) were re-initiated on treatment according to the American Thoracic Society recommendations with a mean of 8 days after drug interruption.

**Conclusion:**

In conclusion, the management of DILI related to anti-tubercular medications in our institution can further be standardized to reintroduce a rifamycin analog with or without ethambutol. Education on the avoidance of concomitant hepatotoxic medications such as acetaminophen and selecting the most appropriate agents to sequentially re-initiate treatment is warranted.

**Disclosures:**

**All Authors**: No reported disclosures.

